# Epigenetic regulation in coronary artery disease: from mechanisms to emerging therapies

**DOI:** 10.3389/fmolb.2025.1548355

**Published:** 2025-01-31

**Authors:** Rui Gao, Meilin Liu, Haoyi Yang, Yuhan Shen, Ni Xia

**Affiliations:** ^1^ Department of Cardiology, Union Hospital, Tongji Medical College, Huazhong University of Science and Technology, Wuhan, China; ^2^ Hubei Key Laboratory of Biological Targeted Therapy, Union Hospital, Tongji Medical College, Huazhong University of Science and Technology, Wuhan, China; ^3^ Hubei Provincial Engineering Research Center of Immunological Diagnosis and Therapy for Cardiovascular Diseases, Union Hospital, Tongji Medical College, Huazhong University of Science and Technology, Wuhan, China

**Keywords:** epigenetics, coronary artery disease, atherosclerosis, DNA methylation, histone modifications, non-coding RNAs, therapeutic targets

## Abstract

Atherosclerosis, the primary cause of coronary artery disease (CAD), remains a leading global cause of mortality. It is characterized by the accumulation of cholesterol-rich plaques and inflammation, which narrow the coronary arteries and increase the risk of rupture. To elucidate this complex biological process and improve therapeutic strategies, CAD has been extensively explored from an epigenetic perspective over the past two decades. Epigenetics is a field investigating heritable alterations in gene expression without DNA sequence changes, such as DNA methylation, histone modifications, and non-coding RNAs. Increasing evidence has indicated that the development of CAD is significantly influenced by epigenetic changes. Meanwhile, the impact of epigenetics in CAD is now transitioning from pathophysiology to therapeutics. Focusing on the key epigenetic enzymes and their target genes will help to facilitate the diagnosis and treatment of CAD. This review synthesizes novel epigenetic insights into CAD, addressing the pathological processes, key molecular mechanisms, and potential biomarkers. Furthermore, we discuss emerging therapeutic strategies targeting epigenetic pathways. By focusing on pivotal enzymes and their associated genes, this work aims to advance CAD diagnostics and interventions.

## 1 Introduction

Cardiovascular diseases have been the leading cause of death worldwide, with coronary artery disease (CAD) being particularly prevalent and imposing significant health and economic burdens (GBD 2021 Causes of Death Collaborators, 2024). Atherosclerosis (AS) is the foundation for CAD and represents a persistent inflammatory condition. It is characterized by the buildup of various immune cells and lipids underneath the coronary endothelium that leads to luminal obstruction (Herrero-Fernandez et al., 2019). Once the endothelial function of the coronary wall is impaired, AS begins due to the accumulation of lipoprotein in the vessel intima. Endothelial cells (ECs) activate and trigger monocyte recruitment from the bloodstream. These monocytes differentiate into macrophages upon migrating into the subendothelial space and subsequently engulf lipids. The proliferation of foam cells results in fatty streaks that develop into atherosclerotic lesions. Vascular smooth muscle cells (VSMCs) are subsequently recruited, proliferate, and synthesize an extracellular matrix, promoting fibrous plaque formation. Ultimately, the encroachment of these plaques on the coronary artery lumen can lead to thrombogenic events (Badimon et al., 2012; Tabas et al., 2015). The progressive course of CAD is influenced by a combination of factors that complicates its management. Despite the existence of numerous clinical therapeutic strategies, the existing scientific knowledge does not fully explain the complex pathophysiological processes of CAD, and the treatment remains challenging (Bansal and Hiwale, 2023). Therefore, the opening up of novel therapeutic directions is a priority for current research.

In recent decades, epigenetics has become a new field of study that can potentially offer alternative strategies for treating human diseases (Portela and Esteller, 2010). Epigenetic changes, defined as heritable alterations in gene expression that do not affect DNA sequences, primarily involve DNA methylation, post-transcriptional histone modifications, and non-coding RNAs (ncRNAs) (Ragunathan et al., 2015). Epigenetic modifications remodel chromatin conformation to control DNA accessibility by modulating gene expression at multiple levels. Crucially, epigenetic processes are reversible and highly sensitive to environmental influences, making them attractive therapeutic targets (Baccarelli and Ghosh, 2012; Majnik and Lane, 2014; Marchio et al., 2019).

The involvement of epigenetics in the initiation and progression of CAD has become a major research focus. It has been reported that epigenetic changes are responsive to environmental risk factors associated with CAD and influence the progress of CAD by controlling the interaction between genes and the environment (Udali et al., 2013). For example, high-risk factors such as obesity cause CAD strongly associated with epigenetic pathways (Smail, 2019). CAD patients who smoke have specific DNA methylation patterns, while quitting smoking can reduce the risk of developing CAD (Steenaard et al., 2015). Besides, significant epigenetic alterations occur in the development of CAD, such as abnormal histone modifications in monocytes from CAD patients and a correlation between levels of circulating miRNA and the presence of CAD (Xiao et al., 2018; Kondracki et al., 2024). More importantly, epigenetic-based therapies may provide a new option for CAD patients. In fact, several existing pharmaceutical agents employed in the treatment of CAD have been shown to act through the modulation of epigenetic pathways. Other novel epigenetic drugs under development have initially shown promise for CAD patients (Bergonzini et al., 2023). Consequently, a comprehensive investigation of the epigenetic mechanisms implicated in the pathogenesis of CAD is crucial to advancing our understanding of its management.

## 2 Overview of epigenetics

**FIGURE 1:**
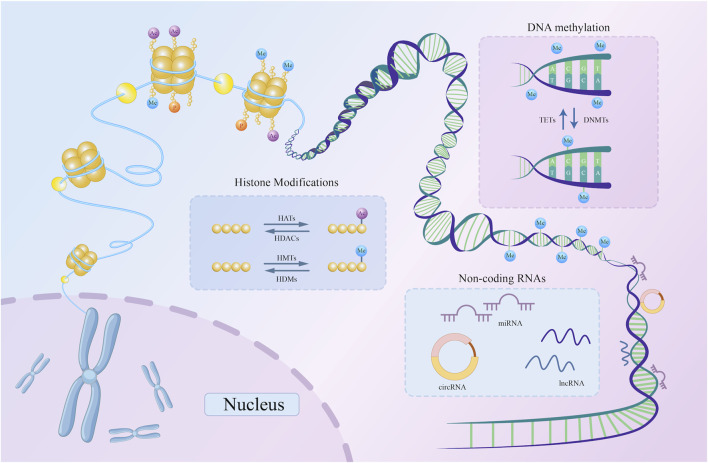
A schematic representation of three basic types of epigenetic regulation. DNMTs catalyze the addition of methyl groups to the 5′-CpG-3′ dinucleotide, while TETs catalyze the removal of methyl groups from the same dinucleotide. Histone acetylation and methylation are examples of histone modifications. HATs and HDACs catalyze the addition and removal of acetyl marks on the histone tail domain, respectively. Similarly, HMTs and HDMs are key enzymes catalyzing histone methylation. By attaching to DNA, non-coding RNAs, including circRNA, lncRNA, and miRNA, can alter the expression of particular genes. DNMT, DNA methyltransferase; TET, Ten-Eleven Translocation; HDAC, histone deacetylase; HAT, histone acetyltransferase; HMT, histone methyltransferase; HDM, histone demethylase; miRNA, microRNA; lncRNA, long non-coding RNA; circRNA, circular RNA; ncRNAs, non-coding RNAs; CpG, cytosine phosphate guanine.

DNA methylation, histone modifications, and non-coding RNA-based regulation are the main epigenetic characteristics of human cells (Egger et al., 2004; Rodenhiser and Mann, 2006; Wilson, 2008). Through epigenetic regulation, active gene states can be stably transmitted across cell generations, exerting a profound influence on cellular function and fate (Kelsey and Feil, 2013). This section provides an overview of these three regulatory mechanisms (Figure 1).

### 2.1 DNA methylation

DNA methylation, regarded as a major epigenetic alteration, has been extensively investigated as a pre-transcriptional modification. This process involves the covalent addition of methyl groups to the fifth carbon atom of cytosine within 5′-CpG-3' (cytosine phosphate guanine [CpG]) dinucleotide, forming 5-methylcytosine (Duan et al., 2016). DNA methyltransferases (DNMTs), such as DNMT1, DNMT3A, and DNMT3B, catalyze this reaction. DNMT3A and DNMT3B are responsible for *de novo* DNA methylation to create new methylation patterns, while DNMT1 maintains existing methylation markers (Chen and Zhang, 2020). Certain demethylating enzymes balance the changes in DNA methylation, such as Ten-Eleven Translocation (TET) enzymes, which remove methylation modifications from DNA by oxidizing methyl groups (Wu and Zhang, 2014).

In the human genome, approximately 70%–80% of CpG dinucleotides are predicted to be methylated. In contrast, demethylated CpGs are locally aggregated into CpG islands, rich in CpG dinucleotides with an uneven distribution. CpG islands typically reside in promoter regions and regulate transcription by modulating transcription factor binding, whereas methylated CpGs are primarily found in non-regulatory parts of the genome (Smith and Meissner, 2013; Greenberg and Bourc’his 2019). Methylation of promoter regions typically reduces accessibility and represses transcription, whereas hypomethylation is associated with active gene expression (Meehan and Stancheva, 2001; Curradi et al., 2002; Weber et al., 2007).

### 2.2 Histone modifications

Histones are alkaline proteins that package DNA into nucleosomes, forming the structural unit of chromatin. Closely linked to the function of DNA molecules as genetic material, histones are classified into five groups: H1/H5, H2A, H2B, H3, and H4. Except for H1/H5, the rest of the histones wrap DNA in an octamer. Each histone consists of an N-terminal tail domain and a C-terminal globular domain, where the tail domain is dynamically modified through acetylation, methylation, phosphorylation, and ubiquitination in response to cellular signals (Bhasin et al., 2006; Draizen et al., 2016). These modifications alter histones’ electrostatic charge and their binding capacity to DNA, contributing to conformational changes in the chromatin structure and influencing RNA transcription levels. Hence, post-translational histone modifications are significant mechanisms underlying gene control (Strahl and Allis, 2000; Berger, 2007; Rothbart and Strahl, 2014). Among these modifications, histone acetylation and methylation are particularly significant and are discussed in detail below.

#### 2.2.1 Histone acetylation

Since they contain several lysine and arginine residues, histones are positively charged. When acetylated on lysine residues, histones drift from DNA as their electric charge is neutralized, lessening the attraction between negatively charged DNA and the histone (Lee et al., 1993). The chromatin structure becomes loose, making it more susceptible to binding to transcriptional regulatory molecules. Therefore, acetylation promotes gene expression (Cheung et al., 2000). Furthermore, the balance of histone modifications is maintained by specific enzyme complexes that can dynamically add or remove these chemical groups (Yang and Seto, 2007). Along with transcription factors and co-regulators that serve as acetylation substrates, histone acetyltransferases (HATs) are recruited to acetylate lysine on histones during acetylation. Correspondingly, histone deacetylases (HDACs) can reverse this alteration by eliminating activating acetyl marks and restoring the positive charge (Blander and Guarente, 2004; Yang and Seto, 2008). The Gcn5-related N-acetyltransferases and p300/CBP families are prominent examples of HATs that are primarily localized in the nucleus. Furthermore, HDACs are classified into subgroups based on their structure and function, including class I HDACs (HDAC1, 2, 3, and 8), class II HDACs (HDAC4, 5, 6, 7, 9, and 10), class III HDACs (SIRT1–7), and class IV (Park and Kim, 2020). Collectively, HATs and HDACs add and remove acetyl groups from the N-terminal tails of lysine residues and are typically related to the activation and suppression of functional gene expression (Kaimori et al., 2016).

#### 2.2.2 Histone methylation

Histone methylation occurs predominantly at the lysine and arginine residues in histones H3 and H4, induced by histone methyl transferases (HMTs) (Wolf, 2009). The functional consequences of methylation are determined by the specific residues modified and the number of methyl groups added. The following methylation residues have been observed in histones H3 and H4: K4, K9, K27, K36, K79, and K20. Among that, H3K4, H3K36, and H3K79 allow transcription, while H3K9, H3K27, and H4K20 are repressive marks (Greer and Shi, 2012).

### 2.3 Non-coding RNAs

According to bioinformatics statistics and the Encyclopedia of DNA Elements (ENCODE) project, approximately 70% of the human genome is transcribed into RNA, whereas only 1%–2% of DNA is directly involved in coding proteins. These protein-coding DNA sequences, known as exons, form the core of genes. In contrast, the remaining genomes that contain non-protein-coding regions, intronic regions, and repetitive sequences are translated into ncRNAs (Harrow et al., 2012). NcRNAs play essential regulatory roles within cells and participate in various biological processes, including gene silencing and activation, RNA splicing and editing, and protein translation (Kaikkonen et al., 2011; Cathcart et al., 2015). According to their size and mechanisms of action, ncRNAs are typically categorized into microRNAs (miRNAs), long non-coding RNAs (lncRNAs), and circular RNAs (circRNAs) (Consortium, 2012; Quinn and Chang, 2016; Aufiero et al., 2019). Here, we provide a concise description of these three ncRNA types.

#### 2.3.1 MicroRNAs

MicroRNAs, approximately 20 nucleotides (nt) in length, have been identified as key regulators of gene expression across various biological networks. Their nucleotide sequences are highly conserved among species, highlighting their evolutionary significance (Friedman et al., 2009). To date, over 2,500 miRNAs have been identified, collectively regulating approximately two-thirds of protein-coding genes (Kozomara and Griffiths-Jones, 2014). miRNAs suppress gene expression by attaching themselves to the 3′untranslated regions of target mRNAs (Carthew and Sontheimer, 2009). Through complementary binding with mRNAs, miRNAs construct extensive regulatory networks that can reversibly adjust gene expression (Pillai, 2005). These miRNA-mRNA interactions are involved in most biological processes, endowing miRNAs with robust and effective regulatory capabilities to influence disease progression (Fabian et al., 2010).

#### 2.3.2 Long non-coding RNAs

LncRNAs were initially considered transcriptional ‘noise' without biological functions. However, recent research has revealed their extensive roles in gene regulation (Ma et al., 2015). Unlike miRNAs, lncRNAs are ncRNA molecules exceeding 200 nt originating from both the coding and non-coding regions of genes. They are expressed in the nucleus or cytoplasm and possibly released into extracellular fluids through extracellular vesicles (EVs) (Okazaki et al., 2002). LncRNAs exhibit high stability throughout an organism’s lifespan and demonstrate complex roles across different pathological states. Exerting their functions by forming RNA-protein interactions, lncRNAs can activate or inhibit pathways through interactions with transcription factors and modulating chromatin-modifying complexes (Quinn and Chang, 2016). In addition, lncRNAs interact with miRNAs in complex regulatory networks. For instance, incomplete base pairing allows miRNAs in RNA-induced silencing complexes to attach to target lncRNAs, which reduces the stability of lncRNA structure and function (Ballantyne et al., 2016). Furthermore, lncRNAs can influence target mRNA expression by competing with miRNAs directly, demonstrating their synergistic role to enhance and diversify biological information regulation (Ebert et al., 2007; Faghihi et al., 2010).

#### 2.3.3 Circular RNAs

CircRNAs, covalently closed-loop molecules, are typically between 100 nt and several kilobases. Predominantly located in the cytoplasm, circRNAs are present across different species (Memczak et al., 2013). CircRNAs often form closed-loop structures through back-splicing processes involving exon, intron, exon-intron, and tRNA intron regions of protein-coding genes (Zhang et al., 2013; Li et al., 2015; Chen et al., 2018). Due to the absence of both a 5′ cap and 3′ end tail, their circular configuration is more resistant to RNase degradation than linear ncRNAs. Certain circRNAs play protein-coding roles that differ from those of ncRNAs (Jeck and Sharpless, 2014). CircRNAs perform diverse functions in gene regulation. For instance, circRNAs interact with miRNAs as competing endogenous RNA (ceRNAs) and interfere with alternative splicing by intervening in looped exons and influencing the splicing patterns of mRNA precursors, resulting in changes in gene expression. CircRNAs can also bind to specific proteins to form complexes that modulate protein function by altering their conformation (Hansen et al., 2013).

## 3 Epigenetic modifications in CAD

Epigenetic alterations in the genome significantly impact the progression of CAD. Exploring the epigenetic mechanisms offers valuable insights into the pathogenesis of disease and aids in its diagnosis and therapeutic strategies. Specifically, epigenetic modifications occur in various cell types throughout the development of atherosclerotic lesions, including endothelial cells (ECs), vascular smooth muscle cells, and macrophages, which play a pivotal role in advancing AS (Grimaldi et al., 2015). Next, we discuss the role of epigenetic modifications in CAD separately in terms of the different cell types mentioned above.

### 3.1 Endothelial cell dysfunction

**FIGURE 2:**
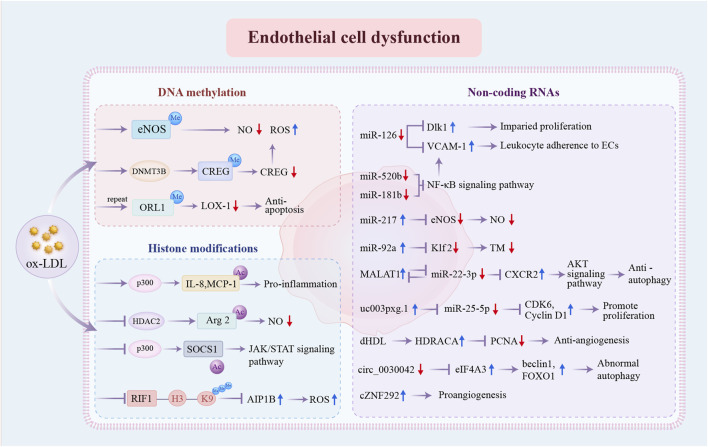
Epigenetic regulatory mechanisms involved in endothelial cell dysfunction. DNA methylation, histone modifications, and non-coding RNAs are the three distinct pathways of pro-atherosclerosis epigenetic regulation in ECs. DNA methylation: ox-LDL promotes DNA methylation at the eNOS promoter and increases DNMT3B expression and CREG promotor methylation. Repeated exposure to ox-LDL causes hypermethylation of OLR1 promoter regions, resulting in decreased LOX-1. Histone modifications: ox-LDL enhances the acetylated status of IL-8, MCP-1, and Arg2 and inhibits the acetylation of SOCS1 through the dysregulation of key enzymes p300 and HDAC2. Repressed H3K9 trimethylation of RIF1 promotes the expression of AIP1B. Non-coding RNAs: the expression levels of multiple non-coding RNAs have been changed, further influencing the expression of specific genes and ultimately resulting in EC dysfunction. ox-LDL, oxidized low-density lipoprotein; eNOS, endothelial nitric-oxide synthase; NO, nitric oxide; ROS, reactive oxygen species; CREG, cellular repressor of E1A-stimulated genes; ORL1, opioid receptor like-1; LOX-1, lipoprotein receptor-1; IL-8, interleukin-8; MCP-1, monocyte chemoattractant protein-1; Arg2, Arginase 2; SOCS1, suppressor of cytokine signaling-1; JAK/STAT, Janus kinase/signal transducer and activator of transcription; RIF1, Rap1 (RAS-related protein 1)-interacting factor 1; AIP1B, ASK1 (apoptosis signal-regulating kinase 1)-interacting protein-1B; Dlk1, delta-like 1 homolog; VCAM-1, vascular cell adhesion molecule 1; ECs, endothelial cells; NF-κB, nuclear factor kappa B; Klf, Krüppel-like factor; TM, thrombomodulin; MALAT1, metastasis associated lung adenocarcinoma transcript 1; CDK, cyclin-dependent kinase; dHDL, dysfunctional high-density lipoprotein; HDRACA, high-density lipoprotein-regulated angiogenesis in coronary artery disease; PCNA, proliferating cell nuclear antigen; eIF4A3, eukaryotic initiation factor 4A-III; FOXO1, Forkhead box O1; other abbreviations are shown in Figure 1.

Endothelial cell dysfunction is recognized as the critical early event in the development of AS (Gimbrone and Garcia-Cardena, 2016). Under normal conditions, ECs generate vasodilator and vasoconstrictor molecules, such as endothelin and nitric oxide (NO), to regulate vascular tone and facilitate vascular repair, maintaining vascular homeostasis. However, different cardiovascular risk factors can trigger and exacerbate homeostasis disruption, resulting in loss of function (Sun et al., 2019; Xu et al., 2021). Figure 2 illustrates the specific epigenetic regulatory mechanisms underlying endothelial cell dysfunction.

#### 3.1.1 DNA methylation

DNA methylation is essential for the synthesis of endothelial nitric-oxide synthase (eNOS). Under physiological conditions, the eNOS promoter exhibits low levels of methylation in ECs, resulting in transcriptional permission. In contrast, ECs exhibit reduced eNOS expression levels, corresponding to DNA hypermethylation at the eNOS promoter in AS (Chan et al., 2004). In addition, the cellular repressor of E1A-stimulated genes (CREG), a vascular protective substance, is markedly downregulated in atherosclerotic vasculatures as oxidized low-density lipoprotein (ox-LDL) increases DNMT3B expression, leading to CREG promoter methylation. This is a novel mechanism that promotes EC dysfunction and the progression of AS (Liu et al., 2020). However, variations in the methylation status of pivotal genes in endothelial cells can also mediate adaptive protection against CAD. The lectin-like oxidized low-density lipoprotein receptor-1 (LOX-1), encoded by the OLR1 gene, is the primary receptor for ox-LDL in ECs and facilitates pro-atherogenic effects (Sawamura et al., 1997). Unexpectedly, after repeated and continuous exposure to ox-LDL, ECs develop resistance to apoptosis due to epigenetic remodeling, which is associated with attenuated pro-apoptotic LOX-1 caused by modifications in the OLR1 promoter’s methylation (Mitra et al., 2011).

#### 3.1.2 Histone modifications

The accumulation of ox-LDL within the vascular vessel wall causes endothelial inflammation and vascular homeostasis imbalance, in which histone modifications affect gene transcription. HDAC2 is less expressed in human ECs because of ox-LDL, resulting in decreased endothelial nitric oxide via increased acetylation levels of Arginase 2 (Arg2) (Pandey et al., 2014). Ox-LDLs also induce specific modifications in interleukin (IL)-8 and monocyte chemoattractant protein-1 (MCP-1) gene promoters by attracting histone acetyltransferase p300 in ECs, causing ECs to release chemoattractants and proinflammatory cytokines (Dje N’Guessan et al., 2009). Meanwhile, regulation by proinflammatory cytokines suppresses RIF1 (Rap1 [RAS-related protein 1]-interacting factor 1)/H3K9 trimethylation-mediated epigenetic processes in ECs. This discovery leads to the identification of a shorter form of AIP1 (ASK1 [apoptosis signal-regulating kinase 1]-interacting protein-1) in diseased aortas of atherosclerotic plaques, with AIP1B augmenting reactive oxygen species (ROS) generation and further leading to EC dysfunction (Li et al., 2020). In addition, the suppressor of cytokine signaling-1 (SOCS1) is a negative mediator of inflammation and can alleviate endothelial injury. Nevertheless, its expression is reduced in CAD patients and *in vitro* because of the dysregulated acetyltransferase p300 (Xue and Deng, 2024). Collectively, these mechanisms lead to the activation of inflammation and EC dysfunction.

#### 3.1.3 Non-coding RNAs

The expression of multiple miRNAs is usually repressed in the endothelium of CAD patients, thereby losing their protective effects. miR-126, which is highly expressed in ECs, regulates vascular inflammation by inhibiting the production of vascular cell adhesion molecule 1 (VCAM-1) (Harris et al., 2008). Disrupted laminar flow in AS reduces endothelial miR-126-5p levels, impairing endothelial recovery by upregulating delta-like 1 homolog (Dlk1), a Notch1 inhibitor (Schober et al., 2014). Additionally, the expression of miR-520b is suppressed in atherosclerotic plaques, which promotes EC inflammation and monocyte-EC communication via activation of the NF-κB p65-VCAM1 axis (Yang et al., 2021). miR-181b also acts as a critical inhibitor of NF-κB signaling, while its activity is impaired in AS (Sun et al., 2014). Conversely, several overexpressed miRNAs facilitate CAD progression. Aging-associated miR-217 is demonstrated to reduce NO production, promoting EC dysfunction and exacerbating AS in proatherogenic apoE^−/−^mice (de Yebenes et al., 2020). In addition, ECs subjected to disturbed flow exhibit increased miR-92a, which is dynamically regulated by shear stress. The transcription factor Krüppel-like factor 2 (Klf2), which is essential for preserving endothelial function, is less expressed when miR-92a is overexpressed (Wu et al., 2011).

LncRNAs also regulate endothelial integrity. The lncRNA metastasis-associated lung adenocarcinoma transcript 1 (MALAT1) is upregulated in patients with unstable angina and protects ECs from ox-LDL-induced endothelial injury, partly by upregulating CXCR2 expression through competitive binding with miR-22-3p (Tang et al., 2015). Likewise, another study verified that EC proliferation was decreased by the genetic ablation of MALAT1 (Michalik et al., 2014). These two studies indicate that the upregulated lncRNA MALAT1 plays a protective role towards the endothelium in CAD. In contrast, patients with CAD have higher levels of the lncRNA uc003pxg.1, which binds to miR-25-5p to promote CAD development by increasing EC migration and proliferation (Li et al., 2021). Normal HDL suppresses the expression of lncRNA HDRACA in ECs to promote angiogenesis. However, when HDL from CAD patients undergoes several changes that cause it to become dysfunctional (dHDL), it loses its capacity to promote angiogenesis due to the presence of the lncRNA HDRACA (Mo et al., 2023).

Circular RNAs are emerging functional molecules in CAD development. Aberrant autophagic mortality of ECs is harmful to atherosclerotic plaques because EC loss encourages lesional clots. circ_0030042 is markedly downregulated in CAD, which promotes ox-LDL-induced abnormal autophagy in ECs and destabilizes atherosclerotic plaques by targeting eukaryotic initiation factor 4A-III (eIF4A3) (Yu et al., 2021). In the vascular endothelium, the hypoxia-induced circRNA cZNF292 is highly expressed and exhibits proangiogenic activity *in vitro* (Boeckel et al., 2015).

### 3.2 Macrophages and imbalanced lipid homeostasis

AS is a persistent inflammatory condition of the artery wall driven by hyperlipidemia. The etiology of AS is heavily influenced by lipid metabolism, particularly cholesterol metabolism and homeostasis. During this process, cholesterol builds up in macrophage-derived foam cells, resulting in lipid accumulation within plaques, a hallmark of early-stage atherosclerotic lesions. Further clarification of the mechanisms leading to abnormal lipid metabolism and foam cell accumulation is vital for preventing plaque formation and rupture (Ouimet and Marcel, 2012).

An imbalance between low-density lipoprotein cholesterol (LDL-C) and high-density lipoprotein cholesterol (HDL-C) significantly contributes to the development of AS (Emerging Risk Factors et al., 2009). Epigenetic regulatory patterns have been identified in genes involved in lipid transport in CAD. ATP-binding cassette A1 (ABCA1) accelerates cholesterol transport from extrahepatic tissues to new HDL particles. The DNA methylation levels of the ABCA1 promoter are elevated in familial hypercholesterolemia, correlating with reduced circulating HDL-C levels and a higher risk of CAD (Guay et al., 2012). Additionally, several ncRNAs affect foam cell production by regulating macrophage cholesterol export via ABCA1. miR-33 and lncRNA growth arrest-specific 5 (GAS5) are highly expressed in CAD macrophages and suppress ABCA1 expression (Rayner et al., 2010; Meng et al., 2020). Furthermore, a novel lncRNA, CHROME, enhances cholesterol efflux and HDL synthesis by inhibiting the effects of a group of functionally similar miRNAs, such as miR-33 (Hennessy et al., 2019). miR-33 also drives lipid droplet accumulation in macrophages via a lysosome-dependent mechanism that represses key autophagy regulators (Ouimet et al., 2017). The scavenger receptor type B1 (SR-BI), predominantly expressed in the liver, serves as an HDL receptor. miR-223 inhibits HDL-C uptake in atheroprone mice by directly targeting and regulating SR-BI expression (Vickers et al., 2014). Similarly, miR-125b impairs macrophage-specific reverse cholesterol transport by targeting SR-BI (Hueso et al., 2022). In addition, miR-148a modulates hepatic low-density lipoprotein receptor (LDLR), a receptor responsible for clearing circulating LDL-C, thereby influencing cholesterol homeostasis (Goedeke et al., 2015). Together, these findings suggest that epigenetic regulation of lipid metabolism genes significantly influences circulating LDL-C and HDL-C levels, thus influencing the risk of AS.

**FIGURE 3:**
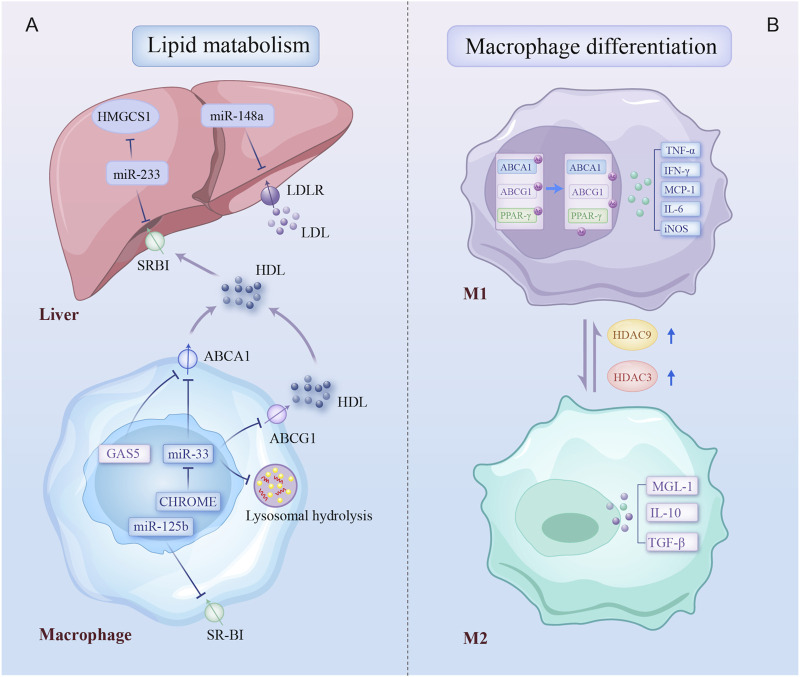
Epigenetic regulatory mechanisms involved in lipid metabolism and macrophage differentiation. Epigenetic modifications regulate two biological processes of macrophage: **(A)** multiple non-coding RNAs contribute to dysregulation of cholesterol production, uptake, and efflux by inhibiting key cholesterol transporters, the cholesterol-producing enzyme, and lysosomal-dependent autophagy processes in liver and plaque macrophage; **(B)** macrophage in atherosclerotic plaques differentiates into a proinflammatory phenotype due to increased expression of HDAC3 and HDAC9. Arrows on specific transporters indicate the direction of transport. HMGCS1, 3-hydroxy-3-methylglutaryl-CoA synthase 1; SR-BI, scavenger receptor type B1; LDLR, low-density lipoprotein receptors; ABCA1, ATP binding cassette subfamily A member 1; GAS5, growth arrest-specific 5; ABCG1, ATP binding cassette subfamily G member 1; CHROME, cholesterol homeostasis regulator of miRNA expression; PPAR-γ, peroxisome proliferator-activated receptor gamma; IFN-γ, Interferon-gamma; TNF-α, tumor necrosis factor-alpha; iNOS, inducible nitric oxidase synthase; TGF-β, transforming growth factor-beta; MGL-1, macrophage galactose-type lectin-1; other abbreviations are shown in Figures 1, 2.

Beyond regulating lipid metabolism, epigenetics regulates macrophage phenotype (Figure 3). HDAC9, abundantly expressed in atherosclerotic macrophages, promotes the inflammatory M1 macrophage phenotype, contributing to AS. HDAC9 deletion reduces inflammation via increasing acetylation of H3 and H3K9 at peroxisome proliferator-activated receptor (PPAR-γ) promoters in macrophages, decreasing AS *in vivo* (Cao et al., 2014a). Similarly, increased HDAC3 expression in atherosclerotic lesions is associated with inflammatory macrophages (Hoeksema et al., 2014). In contrast, ox-LDL-loaded macrophages negatively regulate proinflammatory gene expression at late stages, also regulated by epigenetic mechanisms such as HDAC activity. Macrophages develop the Mox phenotype, a special polarization state that is distinguished by increased production of antioxidant genes under the guidance of nuclear factor erythroid 2-related factor 2 (NRF2) (Jongstra-Bilen et al., 2017). Notably, this reduction in inflammatory response may act as a form of adaptation for foam cells, contributing to a persistent atherogenic state in the lesions. Thus, macrophage polarization is influenced by epigenetic changes, with distinct regulatory patterns emerging at different stages in AS.

### 3.3 Phenotype switch of vascular smooth muscle cells

**FIGURE 4:**
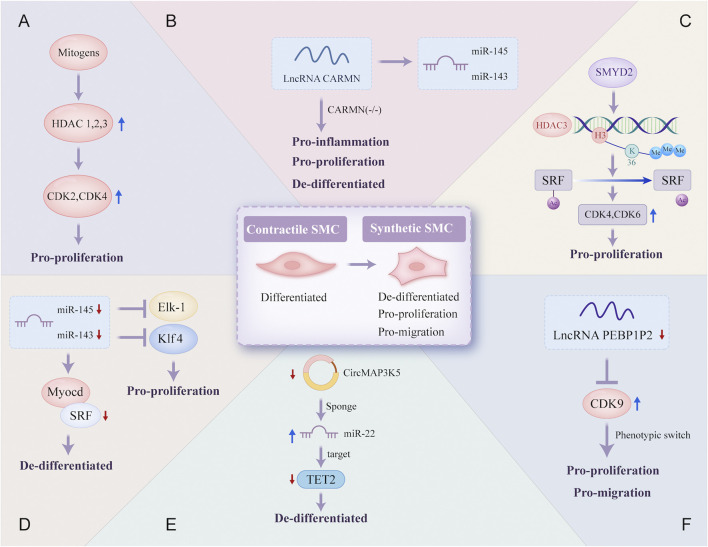
Epigenetic regulatory mechanisms involved in the phenotype switch of vascular smooth muscle cells. Contractile SMC switches to a synthetic phenotype that is de-differentiated, pro-proliferation, and pro-migration in atherosclerosis through a variety of different epigenetic regulatory mechanisms: **(A)** mitogens induce HDAC 1, 2, and 3 transcription in SMC, which promotes SMC proliferation due to increased transcriptional regulation of CDK2 and CDK4; **(B)** lncRNA CARMN can promote the expression of miR-145 and miR-143, and early loss of CARMN controls the phenotype switch of SMC; **(C)** the overexpressed SMYD2 promotes the expression of HDAC3 through H3K36 tri-methylation at its promoter, and then HDAC3 deacetylates SRF, finally promoting SMC phenotypic switching; **(D)** expressions of miR-145 and miR-143 are reduced, which allows the proliferation of VSMC through targeting transcription of SRF, Elk-1 and Klf4; **(E)** CircMAP3K5, significantly downregulated, promotes the de-differentiation of SMC by acting as a TET2-mediated competing endogenous miR-22-3p sponge; **(F)** the decreased expression of lncRNA PEBP1P2 distinctly promotes proliferation, migration, and phenotypic switching in SMC by directly binding to CDK 9. VSMC, vascular smooth muscle cell; Elk-1, ETS-like transcription factor; Myocd, myocardin; SRF, serum response factor; CARMN, cardiac mesoderm enhancer-associated non-coding RNA; CircMAP3K5, circular mitogen-activated protein kinase 5; SMYD2, SET (Suppressor of variegation, Enhancer of Zeste, Trithorax) and MYND (Myeloid-Nervy-DEAF1) domain-containing protein 2; PEBP1P2, phosphatidylethanolamine binding protein 1 pseudogene 2; other abbreviations are shown in Figures 1–3.

In the atherosclerotic lesion microenvironment, VSMCs dedifferentiate and proliferate. This aberrant phenotypic switching of VSMCs is a crucial step in the development of AS (Figure 4).

SMC proliferation after vascular damage is essential for neointimal vessel remodeling. Mounting evidence suggests that histone acetylation is a significant epigenetic alteration in the transcriptional regulation of proliferation-related genes. HDAC, a key regulator of transcriptional cascades, modulates SMC proliferation. A novel epigenetic mechanism reveal that the lysine methyltransferase SMYD2 is overexpressed during injury-induced neointima formation and that SMYD2 promotes the expression of HDAC3 through H3K36 trimethylation at its promoter. HDAC3 directly interacts with and deacetylates the serum response factor (SRF), promoting VSMC phenotypic switching and neointimal hyperplasia (Zhong et al., 2024). Furthermore, inhibiting HDAC activity reduces cyclin D1 and neointima formation, thereby preventing proliferative remodeling caused by intravascular injury, which has important implications for vascular proliferative diseases such as AS (Findeisen et al., 2011).

NcRNAs are also important for VSMC proliferation. miR-143 and miR-145, two of the most abundantly expressed miRNAs in VSMCs under normal conditions, are downregulated in damaged and atherosclerotic regions. Decreased levels of miR-143 and miR-145 may facilitate VSMC proliferation by directly targeting the transcription of SRF. Restoring these miRNAs can prevent smooth muscle hyperplasia associated with vascular damage and AS (Cordes et al., 2009; Elia et al., 2009). CARMN is a lncRNA located upstream of miR-143 and miR-145. CARMN knockout inhibits the expression of miR-143 and miR-145 under normal conditions. Nevertheless, as a microRNA host gene, it has miRNA-independent effects on SMC function. Loss of CARMN controls the functional switch of SMCs towards a pro-atherogenic phenotype and accelerates the development of AS (Vacante et al., 2021). A novel lncRNA, PEBP1P2, suppresses proliferation, migration, and phenotypic switching of VSMCs by interacting with cyclin-dependent kinase (CDK) 9. However, reduced PEBP1P2 levels in the serum of CAD patients indicate a loss of function (He et al., 2020). In addition, circRNAs can regulate gene expression by interacting with specific miRNAs. CircMAP3K5 has been shown to inhibit SMC proliferation in injured arteries by acting as a TET2-mediated competing endogenous miR-22-3p sponge, leading to reduced neointimal formation. Targeting the circMAP3K5/miR-22-3p/TET2 axis may offer a potential therapeutic strategy for AS (Zeng et al., 2021).

### 3.4 Biomarkers

Various epigenetic changes have been identified in multiple cells implicated in the pathogenesis of AS. However, certain molecules that carry epigenetic information have also been detected in the extracellular interstitial space and body fluids. These molecules are predominantly released into body fluids by diseased tissue cells, exhibit functional activity relevant to the disease, and have the potential to serve as promising biomarkers. Numerous epigenetic biomarkers are associated with CAD and its progression, among which potential non-coding RNA biomarkers are listed in Table 1.

**TABLE 1:** Summary of potential non-coding RNAs as biomarkers in CAD.

Types of ncRNAs	Biomarker name	Source	Epigenetic mechanisms	Clinical applications	References
miRNA	miR-126	Plasma	During vascular disease, endothelial miR-126 may be depleted and plasma levels may begin to decline	Plasma miR-126 levels are considerably lowered in CAD patients with elevated LDL levels	Sun et al. (2012)
	miR-126-5p	Plasma	Plasma miR-126-5p involved in the regulation of EC function and plaque formation are markedly decreased in patients with severe CAD	Circulating miR-126-5p may serve as a novel biomarker for predicting the severity and complexity of CAD patients	Li et al. (2016a)
	miR-765, miR-93-5p, miR-433-3p	Plasma	miR-93-5p target ABCA1 gene, while the exact mechanisms for altered expression of miR-765 and miR-433-3p in CHD are still unknown	Overexpressed miR-765, miR-93-5p, and miR-433-3p perform well in predicting CHD severity	Infante et al. (2019)
	miR-223-3p	Plasma	-	Among CAD patients, combining miR-223-3p expression with established cardiovascular risk variables improves prediction of cardiovascular events, particularly stent thrombosis	Pedersen et al. (2023)
	miR-132, miR-140-3p, miR-210	Serum	HIF-1α drives overexpression of miR-210	miR-132, miR-140-3p, and miR-210 may accurately predict cardiovascular mortality	Karakas et al. (2017)
lncRNA	H19	Whole blood	-	Serum H19 is a specific and accurate diagnostic marker for CAD	Xiong et al. (2019)
	CoroMarker	Plasma	Positive correlation between plasma CoroMarker levels and monocyte levels in patients with CAD	CoroMarker, a stable and specific plasma biomarker for CAD, is differentially expressed in plasma of CAD patients	Yang et al. (2015)
circRNA	circ_0001785	Serum	circ_0001785 can minimize EC damage and consequently delay AS via the miR-513a-5p/TGFBR3 ceRNA network	Circulating exosome-derived circ_0001785 shows potential as a biomarker for the treatment of AS.	Tong et al. (2023)
	circNPHP4	PBMCs	circNPHP4 promotes heterogeneous adhesion between monocytes and coronary artery ECs and reduces ICAM-1 and VCAM-1 expression	circNPHP4 overexpression provides a good risk prediction capability for CAD and acts as a possible diagnostic marker for CAD patients	Xiong et al. (2021)

**FIGURE 5:**
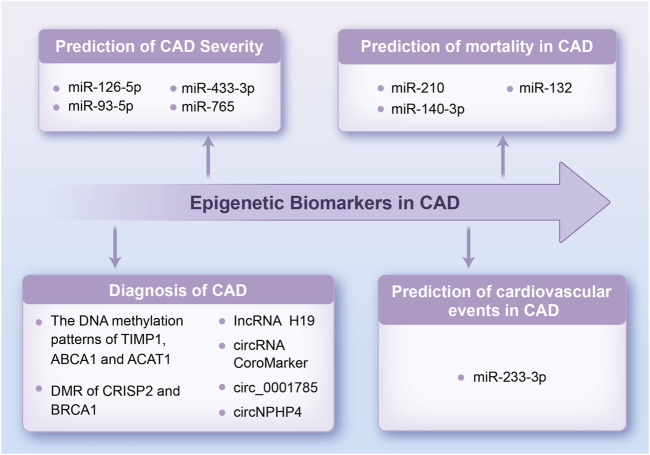
Flowchart of potential clinical applications of epigenetic biomarkers in CAD. This flowchart illustrates the application of representative biomarkers in the diagnosis of CAD, prediction of CAD severity, prediction of cardiovascular events in CAD, and prediction of mortality in CAD. TIMP1, tissue inhibitors of metalloprotease 1; ACAT1, acetyl-CoA acetyltransferase 1; DMR, differentially methylated region; CRISP2, cysteine-rich secretory protein 2; BRCA1, breast cancer 1; other abbreviations are shown in Figures 1–4.

SA, stable angina; CHD, coronary heart disease; PBMCs, peripheral blood mononuclear cells; ceRNA, competing endogenous RNA; ACS, acute coronary syndrome; AS, atherosclerosis; HIF-1α, hypoxia-inducible factor-1, alpha; other abbreviations are shown in Figures 1–5.

The DNA methylation patterns of specific genes related to AS have been proposed as biomarkers of CAD. The DNA methylation patterns of tissue inhibitors of metalloprotease 1 (TIMP1), ABCA1, and acetyl-CoA acetyltransferase 1 (ACAT1) are considerably altered in AS patients and function as biomarkers for the early identification of AS (Ma et al., 2016). Another study analyzed DNA methylation changes on specimens of blood from patients with AS, and differentially methylated regions of cysteine-rich secretory protein 2 (CRISP2) and breast cancer 1 (BRCA1), which regulate endothelial function and angiogenesis, respectively, were found to be the most important DNA methylation biomarkers (Istas et al., 2017). Moreover, cell plasticity in vulnerable plaques is associated with tissue-specific alterations in the dynamic DNA methylation patterns. Since atherosclerotic plaques likely release DNA fragments that carry plaque-specific methylation marks into the circulation, researchers have recently proposed that cell-free DNA methylation (cfDNAme) acts as a non-intrusive and tissue-specific biomarker to provide accurate and rapid information about the condition of atherosclerotic plaques and direct patient risk assessment (Diez Benavente et al., 2024). The first applications of cfDNAme have been reported in cardiovascular diseases and may be applied to CAD patients in the future (De Borre et al., 2023).

In addition, several ncRNAs have been detected in extracellular fluids and circulation. Circulating ncRNAs are remarkably stable and can attach to many types of carrier proteins and exosomes, making them easily detectable. Consequently, ncRNAs may act as important biomarkers for CAD and could potentially serve as new therapeutic targets (de Gonzalo-Calvo et al., 2017; Garcia-Gimenez et al., 2017). miR-126 is a potential biomarker of CAD, with its expression influenced by various CAD risk factors, including aging and dyslipidemia (Fichtlscherer et al., 2010; Sun et al., 2012). In particular, the downregulation of circulating miR-126-5p levels is associated with the severity of CAD. miR-126-5p levels are markedly decreased in CAD patients with multi-vessel disease (Li H. et al., 2016). Similarly, the adjusted multivariate regression model reveals that circulating levels of miR-93-5p, miR-433-3p, and miR-765 are increased in patients with critical coronary stenosis, with a good performance in predicting the severity of CAD. The specific receiver operating characteristic (ROC) curve analysis shows that the areas under the curve (AUC) of circulating miR-765, miR-93-5p, and miR-433-3p expression are 0.762 (p < 0.001), 0.770 (p < 0.001) and 0.771 (p < 0.001), respectively (Infante et al., 2019). Moreover, a recent study suggested that adding miR-223-3p expression to established cardiovascular risk variables such as age, gender, smoking, and diabetes mellitus could enhance the prediction of cardiovascular events in individuals with stable CAD, particularly increasing the predictive value of stent thrombosis (AUC: 0.88 vs. 0.77, p = 0.04) (Pedersen et al., 2023). Individual miRNAs extracted from peripheral blood, particularly miR-210 (AUC: 0.754), miR-140-3p (AUC: 0.756), and miR-132 (AUC: 0.737), can precisely predict mortality in patients with CAD as valuable biomarkers for risk estimation (Karakas et al., 2017). Subsequently, research on lncRNAs in CAD has identified several lncRNAs that could be used as biomarkers for diagnostic and prognostic (Li H. Y. et al., 2016; Arslan et al., 2017). Circulating H19 lncRNA levels in CAD patients are significantly elevated compared with healthy individuals with AUC up to 0.9367 (p < 0.001), making it a potential diagnostic biomarker for CAD (Xiong et al., 2019). Additionally, a novel lncRNA CoroMarker, mainly found in EVs, is a stable, accurate, and unique biomarker of CAD. By constructing a diagnostic model with Fisher’s criteria, the sensitivity and specificity of diagnostic accuracy for CoroMarker alone are 68.29% and 91.89%, respectively (Yang et al., 2015). CircRNAs also are stable and easily detectable, emerging as essential diagnostic tools (Li et al., 2018). Exosome-derived circ_0001785, a novel biomarker of atherogenesis, is reduced in the circulating peripheral blood of CAD patients. It is proved *in vivo* to protect ECs from damage, thereby delaying AS (Tong et al., 2023). Small EV-derived circNPHP4 is significantly upregulated in CAD-related monocytes. ROC analysis performed with circNPHP4 shows high diagnostic performance, as reflected by the Youden index (sensitivity, 87.1%; specificity, 69.7%) (Xiong et al., 2021). These studies highlight the diagnostic and predictive potential of ncRNAs, emphasizing their growing role as prospective biomarkers for CAD. Together, we summarize the clinical applications of these epigenetic biomarkers described above in Figure 5.

To detect these markers, liquid biopsy combined with new molecular analysis techniques is the most promising approach. For cardiovascular diseases, such as CAD, whose affected tissues are not easily accessible, liquid biopsy offers a simple, non-invasive, and reproducible alternative for obtaining dynamic molecular information reflecting the disease state. Typically, liquid biopsies are predominantly performed using blood and are used to analyze a range of biomarkers present in body fluids (Nikanjam et al., 2022; Sharma et al., 2024). As previously mentioned, these CAD-related biomarkers include free extracellular ncRNAs and DNA fragments carrying methylation markers released into the circulation by atherosclerotic plaques. Furthermore, the development of molecular biology techniques, such as polymerase chain reaction (PCR), microarray technology, and high-throughput sequencing, has enabled the acquisition of dynamic molecular information on the state of disease (Xu et al., 2011; Saliminejad et al., 2019; Kojabad et al., 2021). Consequently, in the future, a combination of traditional biomarkers with these newly discovered molecules should be considered in a multi-marker strategy. This could help to monitor disease conditions more accurately, significantly enhance cardiovascular risk management, and guide its treatment.

## 4 Epigenetic treatment for CAD

Epigenetics plays a critical role in CAD by regulating various biological processes at multiple levels, from pathogenesis to potential therapeutic interventions. DNA methyltransferase inhibitors (DNMTi), histone deacetylase inhibitors (HDACi), bromodomain and extra-terminal motif inhibitors (BETi), enhancer of zeste homolog 2 EZH2 inhibitors (EZH2i), and ncRNA-based regulation have been widely studied as potential therapeutics for CAD. Furthermore, the specific functions of several chemicals used in clinical practice can be explained by epigenetic pathways. Table 2 provides an overview of specific drugs targeting epigenetic mechanisms (epidrugs). Epigenetic therapies related to gene regulation may offer novel potential strategies to manage CAD, some of which are already being applied in clinical settings.

**TABLE 2:** Potential epidrugs for the treatment of CAD.

Epigenetics	Classification	Epidrugs	Functional roles	References
DNA methylation	DNMTis	5-Aza-dC	• 5-Aza-dC downregulates expression of inflammation genes (TNF-α, IL-6, IL-1β, and iNOS) and chemotaxis (CD62/L-selectin, chemokine [CCL2/MCP-1], CCL5, CCL9) • 5-Aza-dC inhibits macrophage ER stress • 5-Aza-dC demethylates LXRα and PPAR-γ1	Cao et al. (2014a)
			5-Aza-dC ameliorates abnormal methylation of TET2 promoter, which in turn restores Myocardin expression, thus avoiding excessive VSMC proliferation, migration, and dedifferentiation	Zhuang et al. (2017)
			5-Aza-dC inhibits EZH2-induced downregulation of ABCA1 in macrophages	Lv et al. (2016)
			5-Aza-dC attenuates endothelium dysfunction through restoring CREG expression and activating the p-eNOS/NO pathway	Liu et al. (2020)
		Aza	Aza maintains differentiated SMC phenotype through upregulating PTEN expression	Strand et al. (2020)
			Aza converts naive CD4^+^T cells into functional tregs by inhibiting DNMT1-mediated demethylation of Foxp3-TSDR	Zhu et al. (2024)
		EGCG	EGCG reduces the methylation level of Foxp3-TSDR and increase the levels of anti-inflammatory factors by inhibiting DNMT3B	Zhu et al. (2022)
		RG108	RG108 reverses DNMT3A-mediated transcriptional repression of Klf4	Jiang et al. (2014)
			RG108 regulates DNA methylation status of the PPAR-γ proximal promoter in macrophages through binding directly to DNMT1	Yu et al. (2016)
	Others	Aspirin	Low-concentration acetylsalicylic acid recovers HCAECs function through attenuated methylation of the FGF2 promoter	Chang et al. (2013)
			Lower levels of ABCA1 DNA methylation are linked to acetylsalicylic acid treatment	Guay et al. (2014)
Histone Modifications	HDACi	TSA	TSA prevents the upregulation of ICAM-1, TLR-4, vWF, and ROS, endothelial dysfunction markers brought on by the uraemic environment	Palomo et al. (2020)
			TSA inhibits VSMC proliferation by inducing p21(WAF1) expression and subsequent cell-cycle arrest with decreased Rb protein phosphorylation at the G1-S phase	Okamoto et al. (2006)
			TSA exacerbates AS through the following mechanisms • Increased acetylation at the CD36 promoter • Enhanced the uptake of ox-LDL in macrophage • Elevated TNF-α, SRA and VCAM-1, whereas decreased IL-1β and IL-6	Choi et al. (2005)
		SAHA	SAHA decreases proinflammatory makers, oxidative stress, reactive oxygen species generation, and the extent of atherosclerotic lesions	Manea et al. (2020)
		Romidepsin	Romidepsin promotes VCAM-1 expression through HDAC1/2-mediated inhibition of STAT3 acetylation-dependent GATA6 promoter methylation	Hu et al. (2021)
		TMP195	TMP195 limits proinflammatory responses in macrophages and decreases leukocyte recruitment and endothelial activation, which attenuates lesion formation	Asare et al. (2020)
		RGFP-966	RGFP-966 inhibites the expression of inflammatory cytokines such as TNF-α and IL-1β through HDAC3	Chen et al. (2021)
			RGFP966 enhances acetylation of PPARγ to decrease EC permeability	Zhao et al. (2019)
	BETi	JQ1	JQ1 suppresses cardinal histopathologic features of atherogenesis by controlling rapid inflammatory gene activation via abrogating NF-κB mediated enhancer factor redistribution	Brown et al. (2014); Qi (2014)
		RVX-208	RVX-208 reduces the formation of aortic lesion with an increase in circulating HDL-C and apoA-I and a decrease in LDL-C and hsCRP	Nicholls et al. (2011); Picaud et al. (2013); Jahagirdar et al. (2014); Nicholls et al. (2016); Nicholls et al. (2018); Shishikura et al. (2019); Ray et al. (2020a)
	EZH2i	GSK126	GSK126 increases IGFBP5 expression by suppressing epigenetic silencing of H3K27me3	Xu et al. (2018)
			GSK126 decreases lipid transport and monocyte adhesion mainly by increasing the expression level of ABCA1 and inhibiting VCAM-1 in macrophages	Wei et al. (2021)
	Others	Statins	• Statins reduce the histone modification of MCP-1and IL-8 in endothelial cells, as well as recruitment of CBP 300, NF-κB, and of RNA polymerase II. • Statins partly restore activity of HDAC1 and HDAC2	Dje N'Guessan et al. (2009)
		Resveratrol	Resveratrol pretreatment of endothelial cells reduces eNOS acetylation and SIRT1 levels mediated by oxidants and CSE	Arunachalam et al. (2010)
Non-coding RNAs	miRNAs	miR-33	miR-33 raises the ABCA1 expression in the liver and causes a long-lasting rise in HDL-C levels	Rayner et al. (2011)
	lncRNAs	SNHG12	SNHG12 protects the intima from DNA damage and alleviates AS	Haemmig et al. (2020)
		Dioscin	Through the upregulation of the lncRNA MANTIS, Dioscin stimulates the migration and proliferation of hypoxic endothelial cells	Kong et al. (2021)
	siRNAs	Inclisiran	Inclisiran, a small interfering RNA molecule, reduces LDL cholesterol levels by inhibiting the synthesis of PCSK9	Ray et al. (2019); Ray et al. (2020b)
	ASOs	ANGPTL3-LRx	Oligonucleotides targeting ANGPTL3 retards AS progression and reduces levels of atherogenic lipoproteins: hepatic ANGPTL3 protein, triglycerides, and LDL-C, as well as increases in insulin sensitivity	Graham et al. (2017); Gaudet et al. (2020); Ruscica et al. (2020); Bergmark et al. (2022)
		APO (a)-LRx	Oligonucleotides targeting apolipoprotein(a) reduces Lp(a) concentrations	Viney et al. (2016); Stiekema et al. (2020); Nicholls (2024)
		ApoCIII-LRx	Oligonucleotides targeting ApoCIII leads to a significant improvement in ApoCIII and other atherosclerotic lipid profiles	Alexander et al. (2019); Tardif et al. (2022)

5-aza-dC, 5-aza-2′-deoxycytidine; CCL2, [C-C motif] ligand 2; TSA, Trichostatin A; ER, endoplasmic reticulum; LXRα, liver X receptor α; 5-hmC, 5-hydroxymethylcytosine; PTEN, phosphatase and tensin homolog; SAHA, suberoylanilide hydroxamic acid; PDGF, platelet-derived growth factor; HCAECs, human coronary artery endothelial cells; ICAM-1, intercellular adhesion molecule-1; TLR-4, surface toll-like receptor-4; vWF, von willebrand factor; apoA-I, apolipoprotein A-I; HDL-C, high-density lipoprotein cholesterol; hsCRP, high-sensitivity C-reactive protein; CBP 300 = CREB, binding protein 300; LDL-C, low-density lipoprotein cholesterol; ANGPTL3, angiopoietin-like 3; Lp(a), lipoprotein(a); PCSK9, proprotein convertase subtilisin/kexin type 9; Aza, 5-azacytidine; Tregs, regulatory T cells; Foxp3-TSDR, Forkhead box P3-regulatory T-cell-specific demethylated regions; EGCG, epigallocatechin-3-gallate; EZH2i, enhancer of zeste homolog 2 EZH2 inhibitors; IGFBP5, insulin-like growth factor-binding protein 5; SNHG12, small nucleolar host gene-12; siRNA, small interfering RNAs; ASOs, antisense oligonucleotides; ApoCIII, apolipoprotein C-III; other abbreviations are shown in Figures 1–5.

### 4.1 DNA methylation

Changes in DNA methylation are reversible, providing promising prospects for disease treatment. Research on DNA methylation-based treatments for cardiovascular diseases remains in its early stages. Future studies should focus on identifying potential epidrug targets such as DNMT inhibitors. DNMT inhibitors and similar medications help treat CAD by modulating the methylation and expression of target genes.

5-Aza-2′-deoxycytidine (5-Aza-dC), a DNA methyltransferase inhibitor also known as decitabine, is an anticancer drug that significantly attenuates atherosclerotic lesions. 5-Aza-dC reduces the infiltration of macrophages in AS, which is correlated to decreased expression of inflammatory and chemotaxis genes, as well as suppressed macrophage endoplasmic reticulum stress. Additionally, 5-aza-dC demethylates PPAR-γ1 and liver X receptor α (LXRα) promoters that lead to overexpression of LXRα and PPAR-γ (Cao et al., 2014b). It simultaneously reduces the global 5-methylcytosine content and restores myocardin expression in VSMCs stimulated by platelet-derived growth factors (PDGF), thereby limiting excessive VSMC dedifferentiation and vascular remodeling (Zhuang et al., 2017). Moreover, 5-aza-dC prevents the downregulation of ABCA1 expression (Lv et al., 2016). Recently, it has been demonstrated that 5-aza-dC substantially attenuates ox-LDL-induced EC dysfunction by rescuing CREG expression (Liu et al., 2020). Overall, these mechanisms contribute to 5-aza-dC’s protective effect. Other researchers have also explored the potential of the demethylating drug 5-azacytidine (Aza) as therapy for AS. Aza treatment blocks PDGF-induced SMC dedifferentiation via phosphatase and tensin homolog (PTEN) upregulation, a crucial regulator of the SMC phenotype (Strand et al., 2020). Besides, CD4^+^T cells are induced by Aza *in vitro* to generate induced regulatory T cells (iTregs), adoptive transfer of which attenuates AS due to increasing peripheral blood Tregs and suppressing inflammation. As a demethylating drug, Aza converts naive CD4^+^T cells into functional Tregs by inhibiting DNMT1-mediated demethylation of Forkhead box P3 (Foxp3)-regulatory T-cell-specific demethylated regions (TSDR) (Zhu et al., 2024). Similarly, epigallocatechin-3-gallate (EGCG), a green tea-derived phenol, also leads to a reduction in Foxp3-TSDR methylation levels, which is achieved through the inhibition of DNMT3B. Meanwhile, a notable increase in peripheral blood treg levels is observed, along with elevated transforming growth factor-beta (TGF-β) and IL-10, and a decrease in IL-β and Interferon-gamma (IFN-γ) levels, thereby attenuating atherosclerosis (Zhu et al., 2022).

Different from the above DNMT inhibitors, RG108 is a non-nucleoside analog, showing an inhibitory effect toward DNMT3A. Hemodynamic disturbed flow is considered to be associated with atherosclerosis susceptibility by inducing DNA methylation of the Klf4 promoter in ECs. RG108 reverses this DNMT3A-mediated transcriptional repression of Klf4 and restores its anti-inflammatory protective effects (Jiang et al., 2014). Furthermore, it has been demonstrated that this compound is also capable of binding directly to DNMT1, thereby inhibiting its activity. DNMT1 has been shown to regulate the DNA methylation status of the PPAR-γ proximal promoter in macrophages during the development of AS (Yu et al., 2016). Inhibition of DNMT1 by RG108 could be considered a potential strategy for the treatment of CAD.

Furthermore, there is evidence that certain pharmaceuticals can exert therapeutic effects by modulating the methylation status of genes. For example, aspirin, a standard medication under current rules, has been shown to affect gene methylation. L5, the sole subfraction of LDL, which causes apoptosis in cultured ECs by increasing methylation of the fibroblast growth factor-2 (FGF2) promoter, is significantly increased in patients with CAD. Low-dose aspirin attenuates the detrimental effect of L5 by attenuating this methylation effect (Chang et al., 2013). Aspirin therapy also lowers ABCA1 DNA methylation levels, thereby stimulating reverse cholesterol transport and reducing the incidence of AS and CAD (Guay et al., 2014).

To summarize, a variety of DNMT inhibitors have been extensively studied in the context of CAD, with promising results indicating their potential for treatment. Further in-depth studies are recommended in the future to determine the efficacy of drugs based on DNA methylation modulation in benefitting patients with CAD.

### 4.2 Histone modifications

Among the multiple histone modifications, histone acetylation and methylation are crucial in treating CAD. Key enzymes and functional proteins within these modification processes can be used as therapeutic targets for managing CAD.

#### 4.2.1 HDACi

Trichostatin A (TSA), a potent inhibitor of HDAC, has been extensively researched in the pathogenesis of CAD. TSA prevents the upregulation of EC dysfunction markers induced by the uremic milieu, such as intercellular adhesion molecule-1, von Willebrand Factor, and ROS, whereas EC dysfunction is an early cause of AS in chronic kidney disease (CKD). Therefore, we hypothesized that a specific blockade of HDAC by TSA would prevent CKD-induced AS (Palomo et al., 2020). In addition, TSA inhibits VSMC proliferation by inducing cell cycle arrest, which may attenuate the progression of AS (Okamoto et al., 2006). As a result, TSA has been considered for treating AS and CAD. Nevertheless, the first mouse study on AS treatment with TSA found that it enhanced atherosclerotic lesion formation. TSA increased the acetylation of the CD36 promoter region in macrophages, enhancing the intake of oxLDL. Furthermore, scavenger receptor A (SRA), tumor necrosis factor-alpha (TNF-α), and VCAM-1 were also elevated with TSA treatment, exacerbating AS (Choi et al., 2005). These results show that elevated histone acetylation can affect the progression of AS by altering the expression of ox-LDL receptors and proatherogenic genes. In addition to TSA, treatment with suberoylanilide hydroxamic acid (SAHA), a pan-HDAC inhibitor proved to be one of the antineoplastic drugs, decreased the extent of atherosclerotic lesions in ApoE−/− mice, possibly through a combination of mechanisms that included negative regulation of oxidative stress and proinflammatory marker expression (Manea et al., 2020). Moreover, Romidepsin is another approved HDACi with a unique structure, and it reduces diet-induced atherosclerotic lesion accumulation in Apoe−/−mice through simultaneous inhibition of HDAC1/2 (Hu et al., 2021). In brief, these data suggest that pharmaceutical treatment focused on HDACs is an effective therapeutic approach for AS.

Further studies have shown the role of targeting specific HDACs in AS. Deletion of HDAC9 reduces AS *in vivo* (Cao et al., 2014a). Concurrently, lesion formation was attenuated by blocking HDAC9 with the class IIa HDAC inhibitor TMP195. This blockade limited proinflammatory responses in macrophages and decreased endothelial activation and leukocyte recruitment. Moreover, TMP195 diminishes plaque vulnerability, improving plaque stability in advanced lesions. Monocytes from individuals with established AS produced fewer inflammatory cytokines, including IL-1β and IL-6, when treated *ex vivo* (Asare et al., 2020). Besides, HDAC3 also represents a highly effective target, and the development of small-molecule inhibitors that specifically target this protein is currently a major area of research. HDAC3-selective inhibitor RGFP-966 alleviates atherosclerotic lesions and inhibits endothelial-to-mesenchymal transition in atherosclerotic plaques. *In vitro* studies show that RGFP-966 inhibits the expression of inflammatory cytokines such as TNF-α and IL-1β through HDAC3, thereby suppressing the inflammatory response of ECs (Chen et al., 2021). The inhibition of HDAC3 with RGFP966 has also been demonstrated to enhance acetylation of PPARγ, thus exerting a protective effect against increased endothelial cell permeability (Zhao et al., 2019). These results indicate that subtype-specific targeting of HDACs, especially in macrophages as key immune regulators, may offer a promising approach for AS treatment. Furthermore, a novel method was developed to conjugate an esterase-sensitive motif (ESM) onto HDACs that target monocytes and macrophages expressing carboxylesterase 1 (CES1). These results indicate that ESM-HDACi changes macrophages toward a less severe phenotype by reducing their maturation and proinflammatory responses; however, it is challenging to reverse the plaque size in AS (Luque-Martin et al., 2019).

Therefore, we propose that HDACi can have potential therapeutic implications in AS, and both subtype-specific HDACs and cell-type-specific targets are of significant relevance for future therapy.

#### 4.2.2 BETi

BET proteins facilitate the recruitment of RNA polymerase II to enhance transcription by recognizing and reading acetylated histones. One of the first substances to be created that can bind acetylated histones is the BET bromodomain inhibitor JQ1, which stops BETs from attaching to histone tails and causes transcriptional inhibition. JQ-1 is a broad-spectrum BETi that significantly attenuates endothelial activation, as revealed by suppressing TNF-α induced leukocyte migration and adhesion *in vitro* and *in vivo* (Qi, 2014). Ten weeks of JQ1 treatment suppressed key histopathologic markers of atherogenesis *in vivo*, as it controlled fast inflammatory gene activation through abrogating NF-κB mediated enhancer factor redistribution, facilitating cell state transitions (Brown et al., 2014). Apabetalone, a novel and selective BET bromodomain antagonist (also known as RVX-208), increases apoA-I and preβ-HDL particles, thus reducing the risk of CVD (Picaud et al., 2013). Treatment with apabetalone dramatically decreased the establishment of aortic lesions, accompanied by an increase in circulating HDL-C levels and a decrease in LDL-C levels. Moreover, apabetalone treatment resulted in substantial reductions in circulating adhesion molecules and proinflammatory cytokines, suggesting both lipid-lowering and anti-inflammatory properties (Jahagirdar et al., 2014). Apabetalone has been evaluated in multiple clinical trials focusing on cardiovascular diseases, diabetes, and some cancers. Clinical trials have shown that apabetalone is safe and well tolerated, making it a promising therapy option for CAD (Nicholls et al., 2011; Nicholls et al. 2016; Nicholls et al. 2018; Shishikura et al., 2019; Ray et al., 2020a).

#### 4.2.3 EZH2i

Recent studies suggest that targeted inhibition of histone modifications such as H3K9me2/3 and H3K27me3 may also be an effective therapeutic option in chronic inflammatory diseases. H3K27me3 is catalyzed by the polycomb repressive complex 2 (PRC2), a multi-protein complex of which the EZH2 is the central subunit (Zhang et al., 2018). GSK126, a specific EZH2i, has been shown to possess anti-inflammatory and protective effects in the context of AS. The expression of insulin-like growth factor-binding protein 5 (IGFBP5) is downregulated in human advanced atherosclerotic plaques as a consequence of epigenetic silence by H3K27me3. GSK126 treatment has been demonstrated to increase the expression of this gene, resulting in an anti-inflammatory response in ECs, which in turn has the effect of reducing monocyte adhesion to ECs (Xu et al., 2018). A comparable study has also demonstrated that GSK126 has the capacity to impede the progression of atherosclerosis by diminishing the formation of foam cells in macrophages and the adhesion of monocytes (Wei et al., 2021). In addition to GSK-126, a variety of EZH2 inhibitors are currently in the clinical research stage, but further exploration is required into their application to cardiovascular disease.

#### 4.2.4 Other drugs regulating histone modifications

Statins are clinically applicable HMG-CoA reductases that are essential to the pathogenesis of AS. Statins reduce acetylation of histones H3 and H4, as well as phosphorylation of histone H3 on the promoters of proinflammatory genes like IL-8 and MCP1, which are upregulated by ox-LDL. Statins, therefore, help control atherosclerotic inflammation and partially reverse oxLDL-associated effects, indicating their benefit in reducing cardiovascular risk (Dje N’Guessan et al., 2009). SIRT1, a NAD (+)-dependent deacetylase, regulates endothelial function. Pretreatment of endothelial cells with resveratrol attenuates oxidant- and cigarette smoke extract (CSE)-mediated SIRT1 levels and eNOS acetylation, further ameliorating EC dysfunction (Arunachalam et al., 2010). However, the role of resveratrol in AS and CAD warrants further investigation.

### 4.3 Non-coding RNAs

In the treatment of AS, researchers have focused on several ncRNAs, including miRNAs, lncRNAs, small interfering RNAs (siRNAs), and antisense oligonucleotides (ASOs), which have impacts on fundamental elements involved in the pathogenesis of the disease and may serve as pivotal therapeutic targets.

Since miRNAs are significant post-transcriptional modulators of lipid metabolism, they represent a novel class of therapeutic targets. miR-33a and miR-33b suppress the expression of the cholesterol transporter ABCA1, with their encoding sequences located in the sterol response element-binding protein genes SREBF2 and SREBF1, respectively. Consequently, blocking miR-33a might be a useful strategy for increasing circulating HDL levels and preventing AS. Rayner et al. systematically delivered an anti-miRNA oligonucleotide that targets both miR-33a and miR-33b in African green monkeys to confirm the function of miR-33a/b in lipid regulation. Their results indicated that miR-33a and miR-33b raised the ABCA1 expression in the liver and caused a long-lasting rise in HDL levels. Notably, miR-33 antagonism also suppressed plasma levels of triglycerides linked to very low-density lipoprotein (VLDL) by enhancing the expression of genes related to fatty acid oxidation and downregulating the genes responsible for fatty acid synthesis. These findings established that pharmacologically targeting miRNA-33 is a promising method for treating hyperlipidemia (Rayner et al., 2011). One of the key challenges in utilizing miRNAs as targets for treatment is making sure they are stable and resistant to degradation enzymes. Recent studies have focused on the use of nucleotide and antisense vectors that imitate miRNAs to prevent their degradation or transcriptional blockage. These advances may lead to new therapeutic possibilities (Chistiakov et al., 2016; Chistiakov et al., 2017; Nicorescu et al., 2019). Small nucleolar host gene-12 (SNHG12), an evolutionarily conserved lncRNA, is strongly expressed in vascular endothelium and progressively declines as lesions progress, which negatively corresponds with senescent markers and DNA damage. SNHG12 administered intravenously protects the intima from DNA damage and alleviates AS. These findings imply that SNHG12 plays a role in controlling the repair of DNA damage in the artery wall and may have consequences for states of chronic vascular disease (Haemmig et al., 2020). In addition, dioscin, a compound widely used for treating CAD in China, stimulates the migration and proliferation of hypoxic ECs by overexpressing the lncRNA MANTIS, which provides new insights into its impact on EC angiogenic activity (Kong et al., 2021).

siRNAs are double-stranded RNA molecules that are 20–25 nt in length. They have the capacity to mediate the degradation of complementary mRNAs via the RNA-induced silencing complex (RISC)-dependent pathway (Iwakawa and Tomari, 2022). Inclisiran (ALN-PCSSC), a novel siRNA molecule, can cleave mRNAs encoding the proprotein convertase subtilisin/kexin type 9 (PCSK9). By preventing the synthesis of PCSK9, inclisiran increases LDLR expression in hepatocytes, enhancing the ability of liver cells to clear LDL. In phase I-III clinical trials, inclisiran significantly decreased LDL-C levels by approximately 50% with the convenience of infrequent dosing and showed a comparable decrease in the absolute risk of coronary events. Moreover, inclisiran has a good safety record, with the major adverse effects roughly equal to that of a placebo (Ray et al., 2019; Ray et al., 2020b). The safety profile of inclisiran is also confirmed by subsequent post-marketing pharmacovigilance studies that it is generally consistent with what is currently known from literature data. Among these adverse reactions, injection site reactions and muscle pain are relatively frequent and significant, but no cardiovascular side effects are found (Cicala et al., 2024; Zou et al., 2024). Overall, inclisiran might offer a novel approach to reducing LDL-C and a more effective and safe RNA medication for cardiovascular diseases.

Another type of RNA drugs is mediated by ASOs that have undergone some chemical modifications. Classical ASOs are short, single-stranded DNA-like molecules that enter the target cell and pair with the mRNA of the target gene to form a double-stranded structure that can be degraded by the enzyme Ribonuclease H (Rinaldi and Wood, 2018). Angiopoietin-like 3 (ANGPTL3) has been demonstrated to inhibit the activation of lipoprotein lipase and endothelial lipase, and its absence confers a protective effect in atherosclerosis (Ruscica et al., 2020). AKCEA-ANGPTL3-L_Rx_, known as Vupanorsen, is an ASO targeting hepatic ANGPTL3 mRNA, and randomly assigning both mice and humans to receive this ASO treatment reduced lipoprotein levels and mitigated the progression of AS (Graham et al., 2017). However, while Phase I/II clinical trials demonstrated that Vupanorsen produced a favorable lipoprotein profile and reduced the risk of CAD, development has been halted due to elevated alanine aminotransferase and hepatic steatosis in patients (Gaudet et al., 2020; Bergmark et al., 2022). Notwithstanding, several clinical trials of Vupanorsen are still ongoing, and ANGPTL3 remains a promising target for cardiovascular disease. Similarly, APO(a)-L_Rx_ is an effective treatment for reducing plasma lipoprotein(a) (Lp [a]) concentrations and their associated oxidized phospholipids, LDL-C, and apolipoprotein B-100, by targeting apolipoprotein(a) (Viney et al., 2016). APO(a)-L_Rx_ simultaneously reduced the adhesion promotion of plasma monocytes, hence reducing the cardiovascular risk associated with Lp(a) (Stiekema et al., 2020). A Phase III clinical trial with APO(a)-L_Rx_ is currently underway to determine whether Lp(a) lowering reduces cardiovascular risk (Nicholls, 2024). Furthermore, individuals with apolipoprotein C-III (apoC-III) loss-of-function mutations have been observed to exhibit lower triglyceride levels, as well as an increased risk of CAD. ApoC-III has been identified as a potential therapeutic target for the treatment of atherosclerosis. Some clinical trials have demonstrated that treatment with AKCEA-ApoCIII-L_Rx_ leads to a significant improvement in apoC-III and other atherosclerotic lipid profiles while exhibiting a favorable safety and tolerability profile (Alexander et al., 2019; Tardif et al., 2022).

### 4.4 Challenges and prospects

Even though epigenetic therapies provide a novel therapeutic strategy for CAD, there are some potential issues associated with their implementation in clinical practice. Firstly, non-specific epigenetic modifications or editing of non-target genes may give rise to unintended immune effects. For example, DNMTi and HDACi targeting epigenetic enzymes have a wide range of mechanisms of action and are pleiotropic in normal tissues. When drugs indiscriminately affect multiple members of an enzyme family, they can lead to significant off-target effects and adverse reactions, limiting their clinical application (Shah, 2019). This underscores the necessity for the development of more selective inhibitors that can target individual proteins with greater precision, thereby minimizing deleterious interactions and enhancing their therapeutic efficacy. In addition, the diverse localization and function of epigenetic target enzymes and ncRNAs emphasizes the need for precise delivery of epigenetic drugs to subcellular structures (Chen, 2016; Dang and Wei, 2022). However, current delivery systems face considerable challenges, such as inefficient delivery, insufficient specificity, and potential immune reactions. Therefore, the development of more efficient and safer delivery systems to ensure that epigenetic drugs can precisely reach the corresponding structures in the target cells is key to realizing epigenetic clinical applications (Li et al., 2024). Lipid nanoparticles and novel biomaterials are currently being investigated as targeted delivery vehicles, and these advances may open up new therapeutic possibilities (Liao et al., 2024). Importantly, given the reversibility of epigenetic modifications, the efficacy of the treatment may be contingent upon ongoing administration. This highlights the possibility of resistance to epigenetic-targeted drugs in the long-term application, which is another factor that limits their further application and requires further validation (Paolini and Souroullas, 2024).

Hence, precise localization and efficient delivery of epidrugs, while avoiding off-target toxicity and drug resistance is of paramount importance to enhance the clinical efficacy of these drugs and concomitantly reduce potential side effects. Notably, rapid advances in sequencing technology have enabled the detection of epigenetic abnormalities with increasing efficiency, thereby significantly enhancing our understanding of the heterogeneity of epigenetic modifications across cell types (Gaulton et al., 2023). Besides, drugs targeting epigenetic mechanisms are promising for advancing personalized medicine due to the relationship between epigenetic traits, lifestyle choices, and environmental influences (Cavalli and Heard, 2019). Overall, while significant challenges must be surmounted, the potential benefits for patients suffering from CAD are significant.

## 5 Conclusion

This review presents the results of epigenetic studies in CAD, demonstrating the significant involvement of epigenetic modifications in the development of CAD by modulating gene expression. Here, we describe these specific epigenetic mechanistic changes in the major cell types involved in AS and outline CAD-related epigenetic biomarkers for diagnosis and prognosis. More importantly, we summarize the epigenetic drugs associated with CAD and their potential therapeutic targets. An overview of current epigenetic studies in CAD reveals that DNA methylation and histone acetylation, along with ncRNAs, have garnered significant attention in the context of CAD. However, the role of other histone modifications, such as histone methylation, remains to be fully elucidated and is a promising avenue for future exploration. Furthermore, there has been a growing body of research in recent years that has confirmed the role of various potential epidrugs in the treatment of CAD, with a shift from exploration at the animal level to clinical trials. Nevertheless, there are still many problems that need to be solved in this process, such as off-target effects and difficulties in delivery systems, meaning that the safe and effective application of epigenetic drugs in clinical practice remains a major challenge.

Notwithstanding the comprehensive presentation and discussion of the aforementioned contents, there remain certain aspects that have not been covered and require further exploration. CAD is a multifaceted condition influenced by numerous factors, with epigenetics potentially serving as a bridge between these risk elements and the progression of the disease. Further investigation into the epigenetically mediated mechanisms of diverse cardiovascular risk factors in CAD could facilitate a more comprehensive understanding of disease progression. It is important to note that certain traditional risk factors are associated with sex differences, leading to different correlations with CAD-related adverse events. Not only that, but sex differences also arise from complex interactions between sex chromosomes as well as sex hormones, which lead to differential activation of epigenetic molecular mechanisms that influence CAD and AS phenotypes. Therefore, an expanded discussion of sex-specific differences in the epigenetic regulation of CAD is warranted. Notably, inconsistencies have been observed in epigenetic studies, and at present, an explanation for these divergent results is lacking. Furthermore, multiple epigenetic alterations that have been described may coexist and regulate each other in the identified disease states, but these interactions are not yet fully understood. Consequently, these differences and interactions require further in-depth studies to better understand the function of epigenetics in the development of CAD.

In conclusion, the field of epigenetics has had a profound influence on our understanding of gene regulation in atherosclerotic CAD, and further studies are expected to provide opportunities for the prevention, early diagnosis, and personalized treatment of CAD.
